# Spin-wave duplexer studied by finite-element micromagnetic simulation

**DOI:** 10.1038/s41598-018-34928-0

**Published:** 2018-11-07

**Authors:** Sang-Koog Kim, Hyeon-Kyu Park, Jaehak Yang, Junhoe Kim, Myoung-Woo Yoo

**Affiliations:** 1 National Creative Research Initiative Center for Spin Dynamics and SW Devices, Nanospinics Laboratory, Research Institute of Advanced Materials, Department of Materials Science and Engineering, Seoul National University, Seoul, 151-744 South Korea; 2 Present Address: Centre de Nanosciences et de Nanotechnologies, CNRS, Univ. Paris-Sud, Université Paris-Saclay, 91405 Orsay, France

## Abstract

We conceptually designed a robust nano-scale waveguide structure suitable for potential use as a spin-wave duplexer that allows signal propagation only of selected narrow-band frequencies and duplex transmission in a three-port device comprising a receiver, a transmitter, and their common antenna. The waveguide structure combines three different arms and a circular ring, both made of nanostrip waveguides and a single magnetic material for reliably controllable propagations of spin waves. We attribute the observed duplex transmission of spin waves of narrow pass bands to scattering of spin waves by edge solitons placed at contact areas between the arms and the circular ring. This work proposes the first concept of nano-scale magnonic duplexers operating beyond GHz-frequency ranges.

## Introduction

Nowadays, amid the growing demand for high-speed information processing technologies^1–3^, various device types are being explored. Three-port electronic devices such as isolators^4^, duplexers^5,6^ and circulators^7,8^ are essential for the control of signal propagations in different paths and, thereby, reliable information transmission. For example, a duplexer allows signals from the transmitter port to be sent only to the antenna port, preventing their propagation to the receiver, while signals from the antenna are directed only to the receiver port. Modern duplexers in electronic devices typically operate in several frequency ranges below 1 GHz.

Meanwhile, spin waves, which are collective excitations of magnetizations in ordered magnets, have been studied as information carriers, since they can be transmitted along nano-scale planar-patterned waveguides made of a single magnetic material, offering the advantages of their short wavelengths (of less than 1 μm), higher frequency ranges (higher than 1 GHz), and non-Joule heat dissipation^9,10^. Therefore, there has been much effort devoted to the development of a variety of types of information processing magnonic devices such as logic gates^11–13^, logic circuits^11–13^, filters^14,15^, and multiplexers^5,6^.

Herein we propose a new concept of spin-wave duplexer entailing a specially designed waveguide structure and local magnetization distributions including edge solitons placed on the paths of spin-wave propagations. Our micromagnetic simulation results suggest an optimal magnonic design that may be implemented in future signal-transmission devices.

## Results

### Model geometry

**Figure 1:**
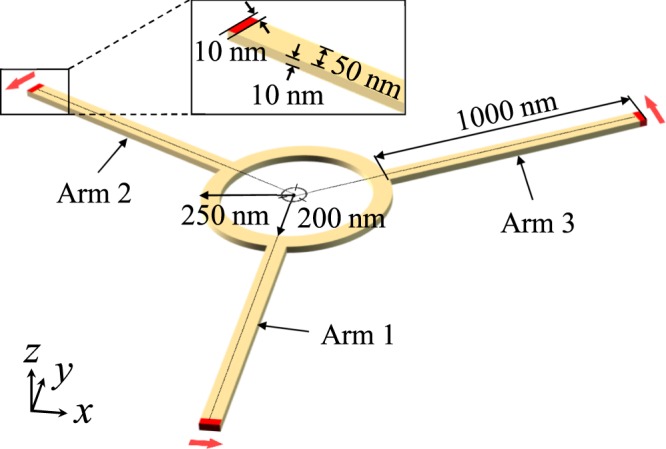
Model geometry. Perspective-view illustration of specially designed geometry for operation of spin-wave duplexer composed of three different arms and circular ring. The waveguide structure is made of Py and 50 nm width and 10 nm thickness. The red-colored end of each arm indicates the region from where spin waves are excited. The dimensions of the spin-wave duplexer are indicated directly in Fig. 1.

Figure 1 illustrates a model structure that consists of a circular ring to which three different arms are attached at 120° angles of separation. This structure is a waveguide for spin-wave propagation that is comprised of Permalloy (Py: Fe_20_Ni_80_) nanostrips of 50 nm width and 10 nm thickness, as indicated in Fig. 1. Spin waves were excited at the end of each arm denoted in red color.

**Figure 2:**
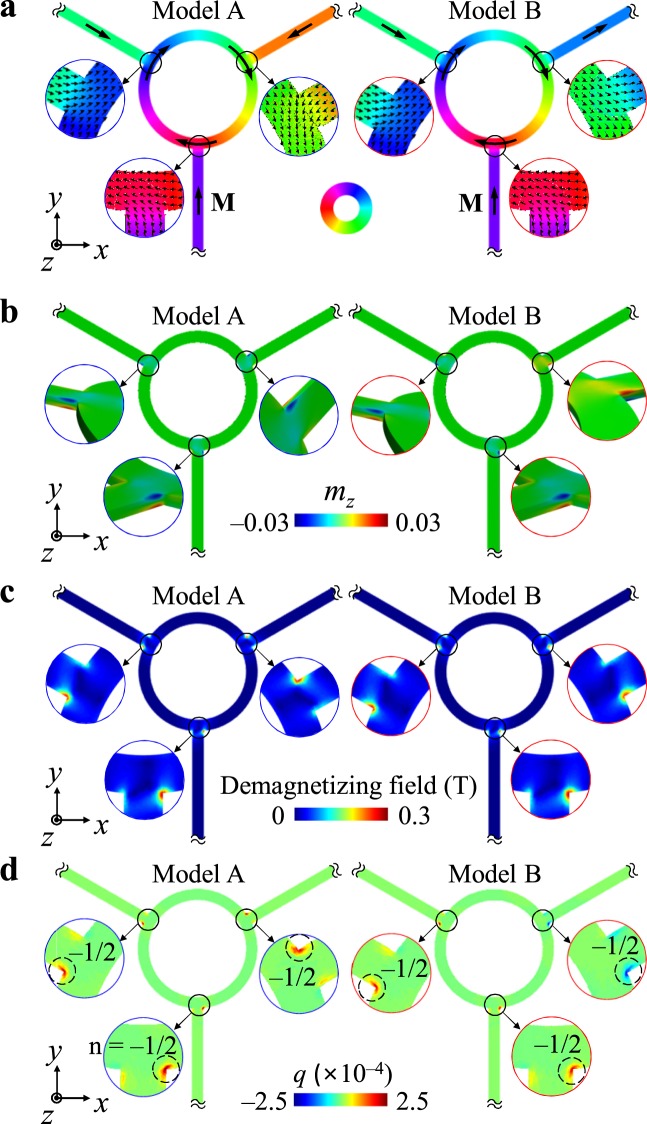
Magnetization configurations. (**a**) In-plane and (**b**) out-of-plane local magnetizations in two different representative ground states, indicated as models A and B. The colors correspond to the in-plane and out-of-plane components of the magnetizations, as indicated by the color bars. (**c**,**d**) display the corresponding demagnetization field and topological charge density distributions, respectively. The insets indicate the corresponding zoomed images along with the winding numbers at the contact areas between the three different arms and the common circular ring.

Figure 2 shows two representative ground-state magnetization configurations among all possible 16 magnetization configurations (see Supplementary Fig. S1) in the model geometry. Overall, the magnetization orientations lie in the longitudinal axis of each arm and the axial direction of the circular ring in order to minimize the dipolar (magnetostatic) energy. In more detail, Model A has inward magnetizations (shown by black arrows on the arms) in all of the arm waveguides toward the circular ring, while Model B has inward magnetizations only in arms 1 and 2 and outward magnetizations only in arm 3. Both models have clockwise spiral magnetizations in the ring. Also, fine features of the in-plane and out-of-plane local magnetizations at the contact areas between the circular ring and the three different arms are highlighted in the zoomed images of Fig. 2(a,b), respectively. A single edge soliton is formed at either side corner of each intersection of the arms and the ring. The local magnetizations in the contact areas result in higher dipolar and exchange energies relative to those in the surrounding areas. Furthermore, spatial distributions of the local demagnetization field and the topological density $q(x,y)=\frac{1}{4\pi }{\boldsymbol{m}}\cdot ({\partial }_{x}{\boldsymbol{m}}\times {\partial }_{y}{\boldsymbol{m}})$^16,17^ are displayed in Fig. 2(c,d), respectively. We also calculated the winding numbers of the edge defects, using $n=-\,\frac{1}{2\pi }{\int }_{\partial {\rm{\Omega }}}\nabla (\theta -{\theta }_{\tau })\cdot dr$^18^, where the integration is restricted to paths ∂Ω along the line boundary of the duplexer geometry, including the edge defects. *θ* is defined as the angle between the local magnetization and an arbitrarily chosen axis and *θ*_*τ*_ is the angle between the tangential direction of the edge boundary of the duplexer. The calculated winding number of each edge defect is noted in the inset of Fig. 2(d), where all the absolute values are less than 1/2, but close to half integer. The total magnetic energies for models A and B are calculated to be slightly different, as E_A_ = 3.16 mJ and E_B_ = 3.10 mJ, respectively, owing to the different positions of an edge soliton in the contact areas between arm 3 and the ring, as clearly seen in Fig. 2(c,d). These configurations are sufficiently stable for spin-wave propagations in a given waveguide and the application of magnetic fields at the end of each arm for spin-wave excitations.

Owing to the three-fold symmetry of the waveguide geometry and characteristic local magnetization distributions, models A and B shown in Fig. 2(a) represent two different characteristic spin-wave propagations. Therefore, only with models A and B, we locally excited spin waves from the end of each arm and allowed them to propagate along each arm and the common circular path. To excite spin waves of a wide range of frequencies, we applied a sinc field given as ${\bf{H}}(t)={H}_{0}\,\sin [2\pi {f}_{0}(t-{t}_{0})]/2\pi {f}_{0}(t-{t}_{0})$ (with *H*_0_ = 100 Oe, *f*_0_ = 50 GHz, and *t*_0_ = 0.01 ns) in the transverse direction of each arm only to a region of 10 × 10 × 50 nm^2^, as indicated by the red color in the inset of Fig. 1.

### Spin-wave propagation for model A

**Figure 3:**
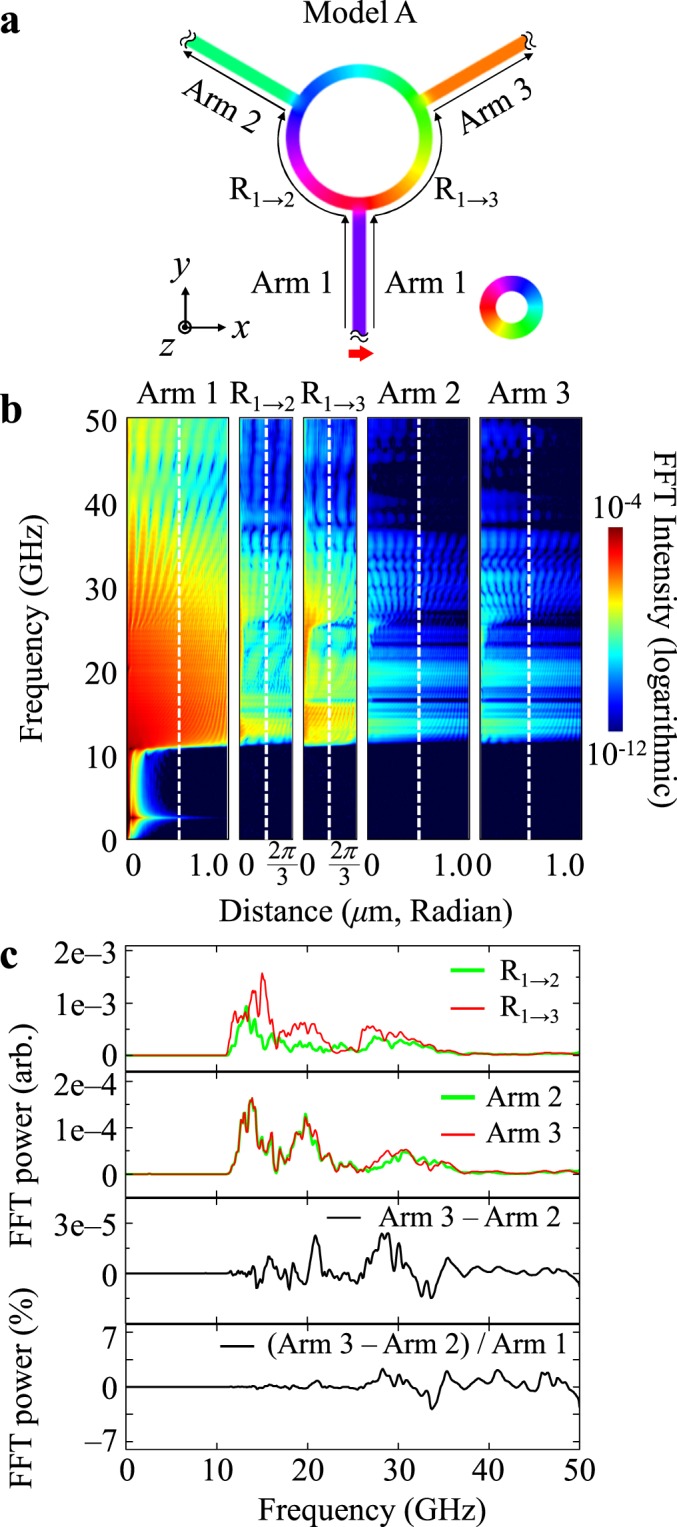
Characteristic behavior of spin-wave propagations for Model A along with spin-wave excitation from end of arm 1. (**a**) Magnetization distribution for Model A and spin-wave excitation from end of arm 1. (**b**) FFTs of *m*_*z*_ oscillations represented on frequency versus distance of each path for model A. (**c**) Each graph shows FFT profiles obtained at the middle of arms and parts of ring (at indicated positions denoted by vertical dashed lines in (**b**)) through which spin waves are propagating.

Since Model A has the three-fold symmetry of the waveguide structure and the local magnetization configurations including edge solitons in the contact areas, the excitation of spin waves at each end of the three different arms and their propagations into the common ring and other arms result in the same dynamic behaviors. Therefore, for Model A, we here show the results for the application of the local magnetic field only to the end of arm 1. In order to interpret the propagation behavior of spin waves, we plotted the FFT powers of the modes’ frequency (*f*) versus the distances of the paths of the three different arms and the circular ring, as shown in Fig. 3(b), along with their line profiles shown in Fig. 3(c). The frequency spectra for the different waveguide paths are separately displayed for arms 1, 2, 3 and the two parts of the ring, denoted as *R*_*a*→*b*_, where *a* and *b* indicate arms 1, 2, and 3, as shown in Fig. 3(a). The FFT power of the spin-wave modes along the straight arm 1 shows a forbidden band as well as a wide pass band for spin-wave propagations typically found along a straight nanostrip of narrow width and thin thickness, as reported in ref.^19^. Spin-wave modes below 11 GHz are not allowed to propagate in such narrow waveguides, owing to the geometric confinement of the nanostrip’s narrow width^19–21^. When the spin waves of a pass band above 11 GHz arrive at the other end of arm 1, i.e., the contact area with the circular ring, they meet two different paths for their further propagation (or transmission), namely R_1→2_ and R_1→3_, as parts of the circular ring waveguide. According to the comparison of the modes’ spectra along the paths R_1→2_ and R_1→3_, the modes spectra are somewhat similar but their FFT powers are contrasting in magnitude in specific pass bands: stronger in R_1→3_ than in R_1→2_. As shown in Fig. 3(c), spin waves of a pass band of 16–18 GHz range are allowed for more propagation along R_1→3_ (red line at top of Fig. 3(c)) than along R_1→2_ (green line). The different degrees of spin-wave propagation along the two different paths of R_1→3_ and R_1→2_ for Model A are caused by the position of the edge soliton at the contact area between arm 1 and the circular ring. As shown in Fig. 2, the position of the edge soliton is closer to R_1→3_ than to R_1→2_, leading to strongly asymmetric localized demagnetization fields in the contact area. The asymmetry of the local demagnetization fields caused by the edge solitons’ position at each contact area can redirect the propagations of spin waves according to their positions^22–25^. The spin wave propagating through magnetic waveguides can be reflected by the non-uniform region of the local magnetizations due to a strongly inhomogeneous internal field, as reported in ref.^22^. For example, the spin waves reaching to the contact area of arm 1 propagate more intensively to the right (R_1→3_) path of the circular ring than to the left (R_1→2_) path, because the asymmetrically localized demagnetization field (i.e., the stronger demagnetization field on the right side of the contact area) scatters more spin waves towards the right path. Then, spin waves, which reach to the contact areas of arm 2 and arm 3, again meet edge solitons placed at different positions around the center of each contact area. Therefore, those spin waves propagate differently along arms 2 and 3. In the contact area of arm 2, the spin waves propagate more intensively towards the arm 2 path than the R_2→3_ path, while in the contact area of arm 3, the spin waves propagate more intensively towards the R_3→2_ path than the arm 3 path. Thus, the mode spectra of arms 3 and 2 become similar, as shown by comparison of their FFT powers in Fig. 3(b). More interestingly, although the spin waves reaching the contact areas of arms 2 and 3 are quite different, the frequency spectra of the spin-wave propagation along them become almost the same. In order to see those effects in better representation, we plotted the FFT powers of the propagating spin waves in the middle of arms 2 and 3 (see second panel in Fig. 3(c)) along with their difference and its normalized value by arm 1, as shown in the bottom of Fig. 3(c). As indicated, the spin-wave propagations in arms 2 and 3 are almost equal.

### Asymmetric propagation of spin-waves by edge solitons

In contrast to the spin-wave propagation behaviors for Model A, Model B showed different characteristic behaviors, due to the different magnetization orientation in arm 3, which is opposite to that for Model A. As shown in Fig. 2, the edge soliton in the contact area of arm 3 is positioned on the right side for Model B, whereas it is on the left side for Model A. The positions of the edge solitons at the other contacts of arms 1 and 2 are the same for models A and B, because the magnetization orientations in those arms are all inwards for both models. Only the different positions of the edge solitons at the contact of arm 3 result in different overall behaviors of spin-wave propagations for Model B relative to those for Model A.

**Figure 4:**
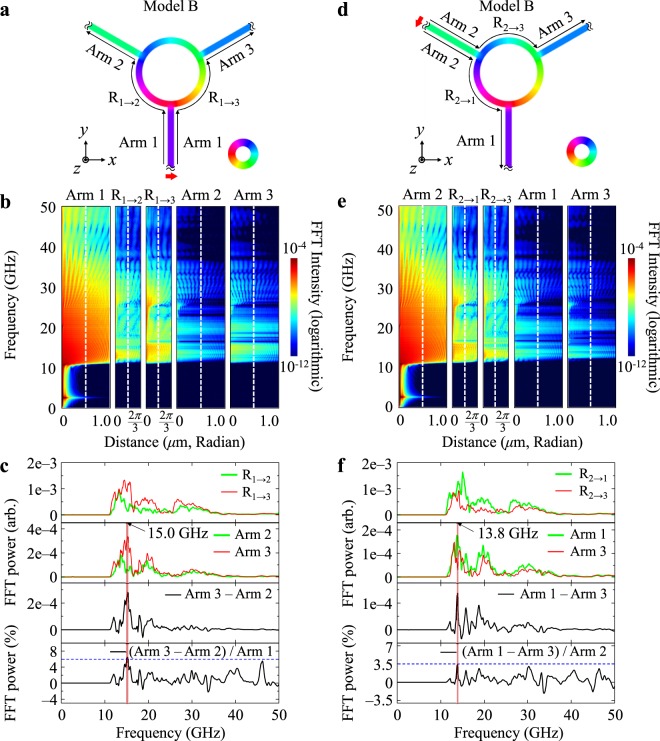
Characteristic behaviors of asymmetric spin-wave propagations for Model B along with spin-wave excitation from ends of arm 1 (left column) and arm 2 (right column). (**a**,**d**) Indicate the magnetization distribution for Model B along with the spin-wave excitation from the end of arm 1 and arm 2, respectively. (**b**,**e**) Indicate the FFTs of the *m*_*z*_ oscillations represented on the frequency versus distance of each path for model B along with the spin-wave excitation from the end of arm 1 and arm 2, respectively. (**c**,**f**) Represent the FFT profiles taken at the middle of the indicated arms and parts of the ring through which spin waves are propagating for spin-wave excitations from the end of arm 1 and arm 2, respectively.

As shown in Fig. 4(a–c), the propagation of spin waves excited at the end of arm 1 is asymmetric for the different paths of R_1→2_ and R_1→3_. The intensity of propagating spin waves is higher along R_1→3_ than R_1→2_. Then, the spin waves that arrive at the contact area of arm 3 propagate more intensively to the right path (arm 3) than to the left path (R_3→2_). Therefore, for Model B, excitations of spin waves at the end of arm 1 yield a higher intensity propagating along arm 3 than along arm 2. Figure 4(c) shows a strong asymmetric propagation of spin waves of 15 GHz between arms 2 and 3 without any manipulation of magnetization configurations by application of local or macro magnetic fields. Promisingly, a certain pass band of around 15.0 GHz can be transmitted with a higher intensity through arm 3, because the position of the edge soliton at the contact of arm 3 leads to more propagation to arm 3 than to R_3→2_.

On the other hand, with the same model B, for the case where spin waves are excited at the end of arm 2, the propagation of spin waves of a pass band of around 13.8 GHz frequency is allowed to propagate more along R_2→1_ than R_2→3_, as shown in Fig. 4(e). Then, the spin waves arriving at the contact areas of arm 1 and arm 3 are transmitted differently to them. Finally, the spin waves of 13.8 GHz are transmitted more through arm 1 than arm 3. Such novel spin-wave propagation behaviors for Model B are technologically useful in duplex transmission of signals by the asymmetric propagation of spin waves at each of the contact areas of arms 1, 2, and 3, depending on the positions of spin-wave excitations. Also, this spin-wave duplexer is promising for operation in higher frequency ranges beyond 1 GHz without Joule heat dissipation^9,10^. According to the asymmetric propagations of spin waves excited from the end of either arm 1 or arm 2, the three different arms can act as an antenna, a transmitter, and a receiver in such a three-port device^4–8^. Although spin waves excited from arm 3 can be transported more through arm 1 and less through arm 2 (not shown here), the excitation of spin waves from the end of arm 3 is not necessary, because arm 3 must act as a signal receiver for the duplexer function.

Figure 4(f) presents which pass band is appropriate for the asymmetric spin-wave propagation through the different paths for spin-wave excitation from the end of arm 2. For the case shown in Fig. 4(a), the highest asymmetry of spin-wave propagation between the path of arm 1, R_1→3_ and arm 3 and that of arm 1, R_1→2_ and arm 2, was obtained with 15 GHz, but for the case shown in Fig. 4(d), it was 13.8 GHz for the different paths of arm 2, R_2→1_ and arm 1 and arm 2, R_2→3_ and arm 3.

### Duplexer function

**Figure 5:**
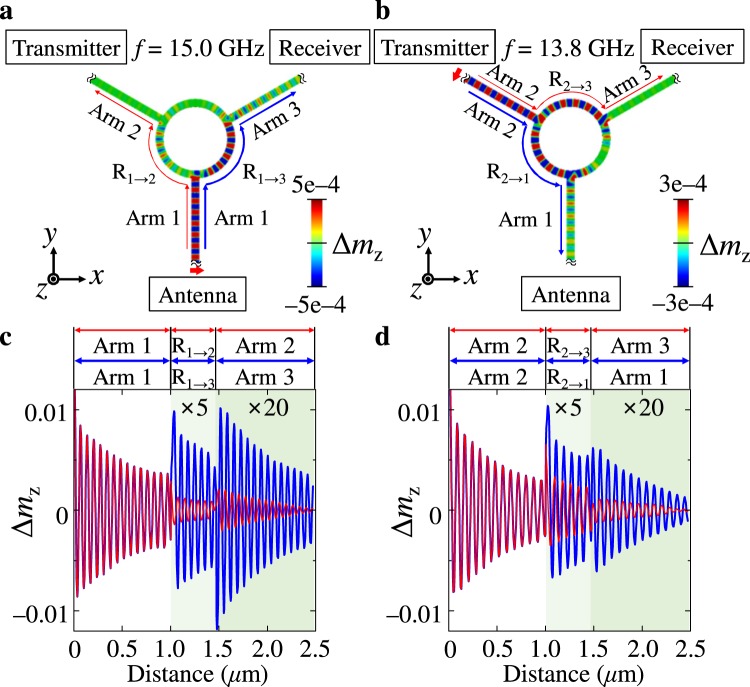
Demonstration of duplexer function. (**a**,**b**) Represent snapshot images of the *m*_*z*_ oscillations taken at 5.0 ns after the excitation of single harmonic frequency spin waves of 15.0 GHz from the end of arm 1, and 13.8 GHz from the end of the arm 2, respectively. (**c**,**d**) Indicate the corresponding *m*_*z*_ variation profiles along the distance of the indicated different paths.

In order to clarify and confirm the function of a duplexer based on spin waves using the specially designed waveguide structure and the magnetization configuration of Model B (see Figs 2 and 4), we conducted further micromagnetic simulations, exciting spin waves of single harmonic frequencies by applying oscillating fields of amplitude *H*_0_ = 100 Oe in a time period of 5.0 ns to the end of arm 1 with *f* = 15.0 GHz (Fig. 5(a)) and arm 2 with *f* = 13.8 GHz (Fig. 5(b)). Snapshot images of *m*_*z*_ (=*M*_*z*_/*M*_S_) variations on the waveguide are shown along the distances of different paths for cases of spin-wave excitations at arm 1 and arm 2 in Fig. 5(a,b), respectively. Also, profiles for the *m*_*z*_ oscillation of spin waves propagating along the different paths are shown in Fig. 5(c,d) for spin-wave excitations at arms 1 and 2, respectively. For the excitation of spin waves from the end of arm 1 (antenna), a higher intensity of propagating spin waves was obtained along the path of arm 1, R_1→3_, and arm 3, while for the excitation of spin waves from the end of arm 2, (transmitter), it was obtained along the path of arm 2, R_2→1_, and arm 1. This result indicates that the spin-wave signals from the antenna port (arm 1) were allowed to propagate to the receiver port (arm 3) for the 15 GHz spin-wave signal, and that the signals from the transmitter port (arm 2) were directed to the antenna port (arm 1) for the 13.8 GHz spin-wave signal, definitively representing the function of a spin-wave duplexer. The duplexer function means that the receiver port does not send signals but only receives them. This specially designed waveguide and the local magnetization configurations precisely demonstrate the duplexer function whereby spin-wave signals of 13.8 GHz from the transmitter are allowed to propagate through the antenna, while spin-wave signals of 15 GHz from the antenna are allowed to transport through the receiver.

## Discussion

In studies thus far, steering spin waves^25–30^ have been operated in two steps: predefining of the spin-wave path, followed by flowing of excited spin waves along it. In such cases, in order to realize a spin-wave duplexer, receiver and transmitter signal paths must be defined repeatedly whenever the signal is received or transmitted. However, our proposed geometry and asymmetric propagations of spin waves of specific pass bands into two different paths entirely relies on the ground-state magnetization configuration specifically set to the model geometry in our simulation study. Also, our proposed concept allows for simultaneous transmission of signals of slightly different pass bands (here 13.8 and 15 GHz in the given dimensions) in sending to and receiving from a common antenna. The specific pass band of spin-wave propagations in such waveguides can be modified according to the dimensions (thickness and width) and the geometry of planar nanostrip waveguides.

Edge solitons and their positions^18,31,32^ at contact areas between the three arms and the circular ring lead to asymmetric propagations of spin waves along different paths according to the underlying physics of scattering such as transmission, reflection, and refraction. It has been reported that magnetic topological defects such as vortex^33^, skyrmion^34^ and domain walls^35^ in nanostrips can interact with spin waves; thus too, the propagation, reflection, and refraction of spin waves can be affected by topological defects such as edge solitons. In our study, edge solitons at the contacts of the three different arms and the common ring led to strongly localized demagnetization fields^22–25^ due to magnetization fluctuations affected by the relative magnetization orientations between each arm and the ring. The localized demagnetization fields are asymmetric (see Fig. 2) at the contact areas through which spin waves pass. The asymmetric demagnetization fields redirect (scatter) spin waves at each of the three contact areas into the two separate paths along which spin waves propagate further. However, a scattering theory for understanding of their behaviors and further experimental demonstration of reproducible control of spin wave propagations in real systems are necessary. For example, for duplexer operation, it is essential to find ways to control the chirality of the ring and the magnetization orientations in the different arms.

In conclusion, our micromagnetic simulation results establish that a spin-wave waveguide composed of three arms and a circular ring enables duplex transmission of spin-wave signals of selected narrow pass bands beyond 1 GHz. This behavior is attributed to asymmetrically distributed local demagnetizing fields in the contact areas, which fields originate from non-uniform magnetizations affected by the relative magnetization configurations between each arm and the circular ring. This concept can further accelerate the miniaturization of a three-port, beyond-GHz-range magnonic duplexers and their real implementation in higher-frequency information processing devices without Joule heating dissipation.

## Methods

### Micromagnetic simulations

In order to demonstrate the function of a spin-wave duplexer using the model geometry, the FEMME code^36^ incorporating the Landau-Lifshitz-Gilbert (LLG) equation^37,38^ was used to conduct finite-element micromagnetic simulations on the curved geometry. The LLG equation is expressed as $\partial {\bf{M}}/\partial t=-\,\gamma ({\bf{M}}\times {{\bf{H}}}_{{\rm{eff}}})+\alpha /|{\bf{M}}|({\bf{M}}\times \partial {\bf{M}}/\partial t),$ where **H**_eff_ is the effective field including the exchange, dipolar and Zeeman interactions. We utilized the following magnetic parameters corresponding to Py: saturation magnetization *M*_S_ = 8.6 × 10^5^ A/m, exchange stiffness constant *A*_ex_ = 1.3 × 10^−11^ J/m, and Gilbert damping constant *α* = 0.01, with zero magnetocrystalline anisotropy. All possible ground-state magnetizations were obtained via free relaxation from the corresponding intended local magnetization distributions in the geometrical confinement. The 3D volume of the duplexer structure was discretized into 148,924 tetrahedrons with an average mesh size of ~5.3 nm. In the present simulations, we recorded the variation of the local magnetizations at 10 ps intervals for 10 ns so as to obtain a high temporal resolution necessary for reliable analysis and interpretations of spin-wave propagations.

## Electronic supplementary material

Supplementary information
