# Supplementary material for: Spin-wave duplexer studied by finite-element micromagnetic simulation
Source: Sci Rep. 2018 Nov 7;8:16511. doi: 10.1038/s41598-018-34928-0 (PMC6220281; doi:10.1038/s41598-018-34928-0)
Supplement: Supplementary file 1 — Supplementary information [file 41598_2018_34928_MOESM1_ESM.doc]

**SUPPLEMENTARY INFORMATION**

**Spin-wave duplexer**

**studied by finite-element micromagnetic simulation**

Sang-Koog Kim^a)^, Hyeon-Kyu Park, Jaehak Yang, Junhoe Kim, and Myoung-Woo Yoo^b)^

*National Creative Research Initiative Center for Spin Dynamics and Spin-Wave Devices, Nanospinics Laboratory, Research Institute of Advanced Materials, Department of Materials Science and Engineering, Seoul National University, Seoul 151-744, Republic of Korea*

^a)^ Correspondence and requests for materials should be addressed to S.-K.K. ([sangkoog@snu.ac.kr](mailto:sangkoog@snu.ac.kr)).

^b)^ Current address: Centre de Nanosciences et de Nanotechnologies, CNRS, Univ. Paris-Sud, Université Paris-Saclay, 91405 Orsay, France


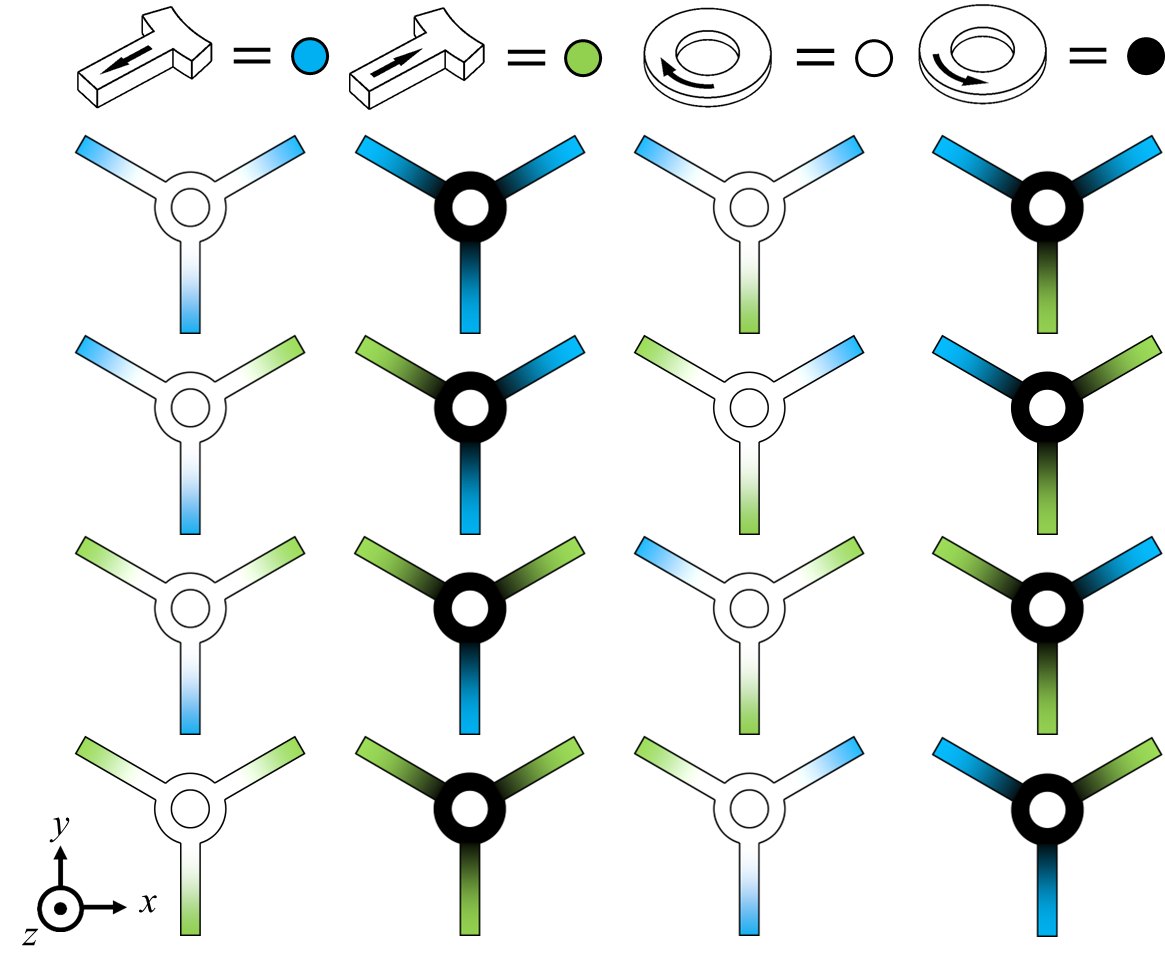


**Supplementary Fig. S1.** Sixteen possible configurations of local magnetizations in three different arms and ring, which were intended before relaxation to find metastable magnetization configurations in 3-port waveguide structure


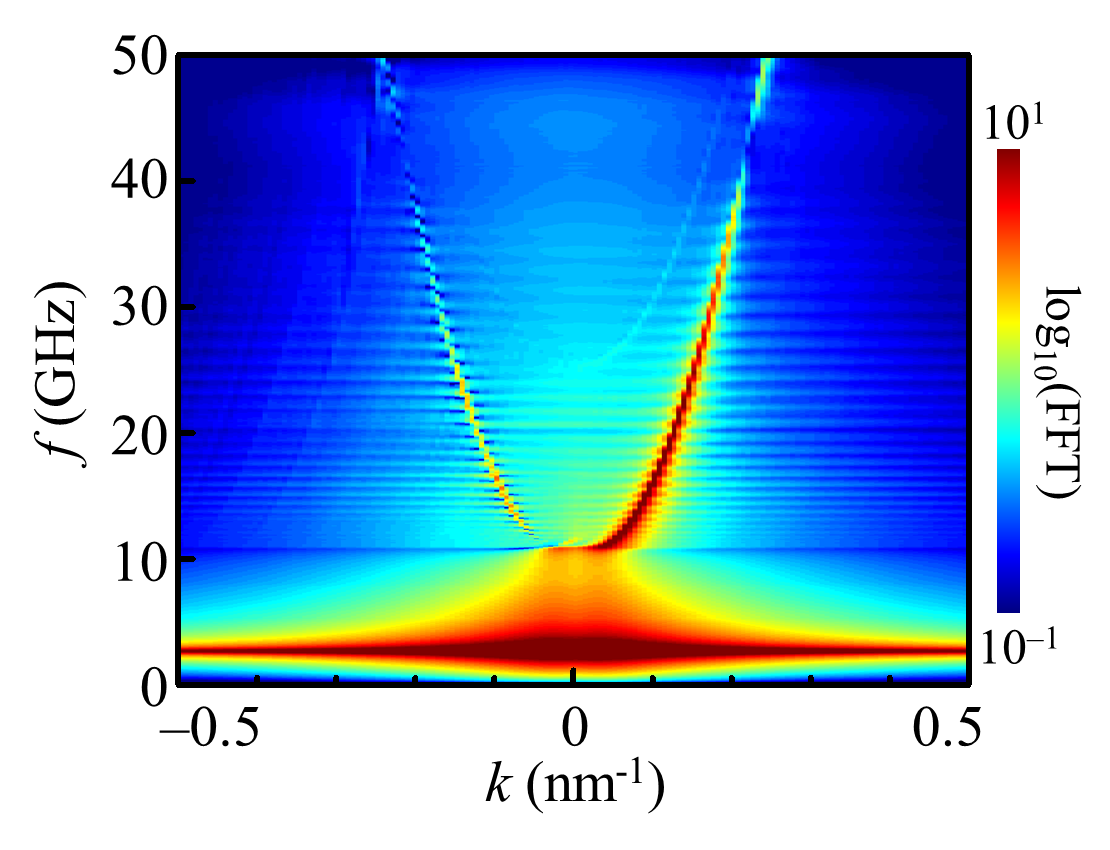


**Supplementary Fig. S2.** Dispersion curve of spin waves propagating along arm 1, after excitation from end of arm 1


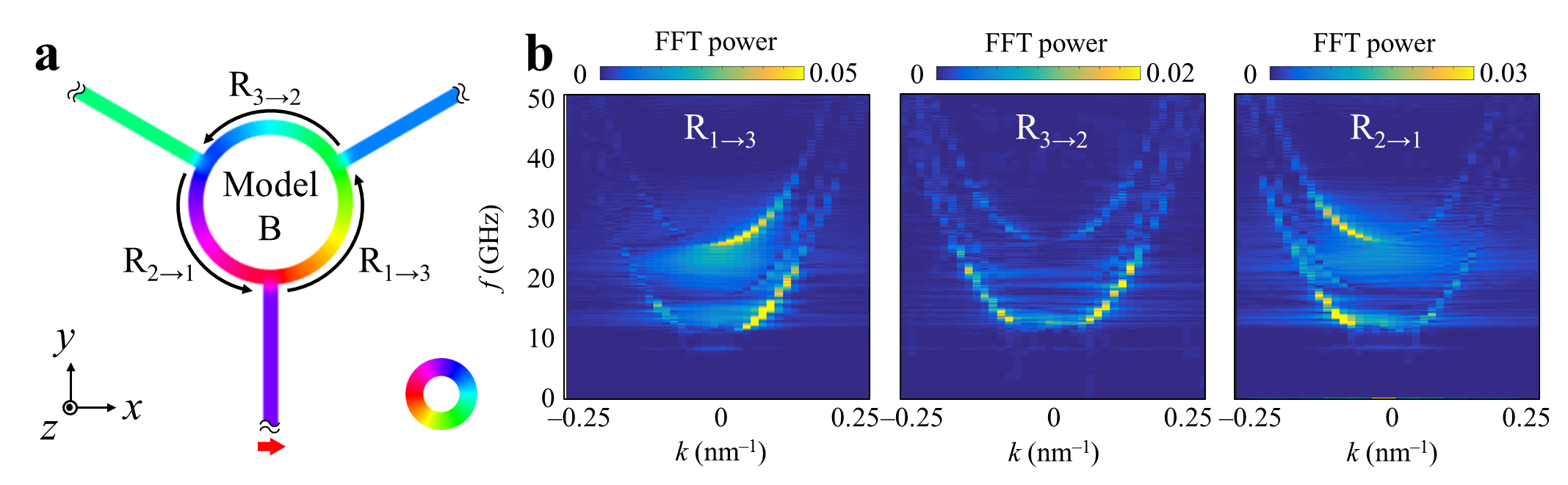


**Supplementary Fig. S3.** (a) Model B for case of excitation of spin waves from end of arm 1. (b) Dispersion curves of propagating spin waves for three different paths of R_1→3_, R_3→2_, and R_2→1_, as indicated. The positive *k* values correspond to the wave vectors of spin-waves propagating forward in the counter-clockwise rotation sense.
